# Computational methods for predicting genomic islands in microbial genomes

**DOI:** 10.1016/j.csbj.2016.05.001

**Published:** 2016-05-07

**Authors:** Bingxin Lu, Hon Wai Leong

**Affiliations:** Department of Computer Science, National University of Singapore, 13 Computing Drive, Singapore 117417, Republic of Singapore

**Keywords:** Pathogenicity islands, Sequence composition, Genome segmentation, Comparative genomics, Outlier detection

## Abstract

Clusters of genes acquired by lateral gene transfer in microbial genomes, are broadly referred to as genomic islands (GIs). GIs often carry genes important for genome evolution and adaptation to niches, such as genes involved in pathogenesis and antibiotic resistance. Therefore, GI prediction has gradually become an important part of microbial genome analysis. Despite inherent difficulties in identifying GIs, many computational methods have been developed and show good performance. In this mini-review, we first summarize the general challenges in predicting GIs. Then we group existing GI detection methods by their input, briefly describe representative methods in each group, and discuss their advantages as well as limitations. Finally, we look into the potential improvements for better GI prediction.

## 1 Introduction

*Lateral gene transfer* (LGT) is the transfer of genes from one organism to another in a way that is different from reproduction. Its ability to facilitate microbial evolution has been recognized for a long time [1]. Despite the ongoing debate about its prevalence and impact [2], [3], [4], the accumulation of evidence has made LGT widely accepted as an important evolution mechanism of life, especially in prokaryotes [5], [6]. As a result of LGT, recipient genomes often show mosaic composition, in which different regions may have originated from different donors. Moreover, some DNA sequences acquired via LGT appear in clusters. These clusters of sequences were initially referred to as *pathogenicity islands* (PAIs) [7], which are large virulence-related inserts present in pathogenic bacterial strains but absent from other non-pathogenic strains. Later, the discoveries of regions similar to PAIs but encoding different functions in non-pathogenic organisms lead to the designation of *genomic islands* (GIs) [8]. GIs are then found to be common in both pathogenic and environmental microbes [9].

**Fig. 1:**
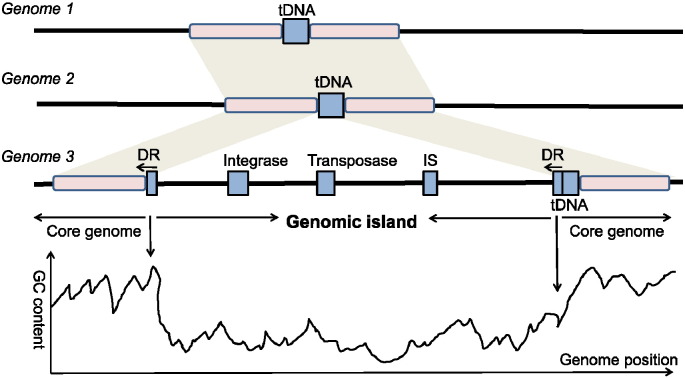
The schematic representation of several GI-associated features. A GI is often absent in closely related genomes. It may also have atypical compositional characteristics compared with the core genome, such as lower GC content. The presence of several sequence elements is indicative of a GI: flanking conserved regions, DRs, insertion sequence (IS) elements and mobility-related genes encoding integrase and transposase.

Specifically, a GI is a large continuous genomic region arisen by LGT, which can contain tens to hundreds of genes. The size of known GIs varies from less than 4.5 kb to 600 kb [3]. Laterally acquired genomic regions shorter than a threshold are also called *genomic islets*[10], [11]. GIs often have phylogenetically sporadic distribution. Namely, they are present in some particular organisms but absent in several closely related organisms. As shown in Fig. 1, GIs have several other well-known features to distinguish them from the other genomic regions [10], [12], [13], such as different sequence composition from the core genome, the presence of mobility-related genes, flanking direct repeats (DRs), and specific integration sites. For example, tDNA (tRNA or tmRNA gene) is well known as a hotspot for GI insertion [11], [14]. However, not all these features are present in a GI, and some GIs lack many of these features. As a consequence, GIs were also considered as a superfamily of mobile elements with core and variable structural features [15].

In addition to the restricted GI definition in [16], GIs are often seen as a broad category of mobile genetic elements (MGEs) [17]. They can be further grouped into subcategories by mobility: some GIs are mobile and hence can further transfer to a new host, such as integrative and conjugative elements (ICEs), conjugative transposons and prophages; but other GIs are not mobile any more [10]. GIs can also be classified by the function of genes within as follows: PAIs with genes encoding virulence factors; *resistance islands* (REIs) with genes responsible for antibiotic resistance; *metabolic islands* with genes related to metabolism; and so on [9]. However, the latter classification may not be definite since the functions of genes within GIs may not be clear-cut in practice.

GIs play crucial roles in microbial genome evolution and adaptation of microbes to environments. As part of a flexible gene pool [18], the acquisition of GIs can facilitate evolution in quantum leaps, allowing bacteria to gain large numbers of genes related to complex adaptive functions in a single step and thereby confer evolutionary advantages [9], [10]. Remarkably, the genes inside GIs can influence a wide range of important traits: virulence, antibiotic resistance, symbiosis, fitness, metabolism, and so on [9], [10]. In particular, PAIs can carry many genes contributing to pathogen virulence [12], [13], [19], and potential vaccine candidates were suggested to locate within PAIs [20]. Thus, the accurate identification of GIs is important not only for evolutionary study but also for medical research.

GIs can be predicted by either experimental or computational methods. Herein, we focus on the in silico prediction of GIs: given the genome sequence of a query organism, identify the positions of GIs along the query genome via computer programs alone. Additional input information may also be incorporated, such as the genomes of other related organisms, and genome annotations.

Langille et al. [17] gave a comprehensive review of GI-related features and different computational approaches for detecting GIs. Recently, in 2014, Che et al. [21] presented a similar review for detecting PAIs. Here, we want to provide an up-to-date review of representative GI prediction methods in an integrative manner. Firstly, we highlight the general challenges in predicting GIs. Then, we subdivide existing methods based on input information, and describe their basic ideas as well as pros and cons. We also propose the promising directions for developing better GI detection methods.

## 2 Challenges in GI prediction

It is a non-trivial task to find laterally transferred regions of relatively small size in a long genome sequence. Two prominent challenges in GI prediction are the extreme variation of GIs and the lack of benchmark GI datasets.

### 2.1 The extreme variation of GIs

It seems easy to predict GIs given the various well-characterized features associated with it. However, the mosaic nature and extreme variety of GIs increase the complexity of GI prediction [3]. The elements within a GI may have been acquired by several LGT events (probably from different origins) and are likely to have undergone subsequent evolutions, such as gene loss and genomic rearrangement [9]. Consequently, the composition, function and structure of GIs can show various patterns. This can be illustrated by GIs in the same species [22], GIs in Gram-negative bacteria [12], and GIs in both Gram-positive and Gram-negative bacteria [12], [15]. The diversity of GIs prevents an effective way of integrating multiple features for prediction. Choosing only a few features as predictors may discard lots of GIs without those features. Even if the fundamental property of GIs, the lateral origin, can be used for prediction, it is still challenging since LGT itself is difficult to ascertain [23].

### 2.2 The lack of benchmark GI datasets

There are still no reliable benchmark GI datasets for validating prediction methods or supervised prediction. With more GIs being predicted and verified, several GI-related databases have been deployed and regularly updated, such as Islander [24], PAIDB [25], and ICEberg [26] (Table 1). However, these databases are mainly for *specific kinds* of GIs, such as tDNA-borne GIs (GIs inserted at tRNA or tmRNA gene sites), PAIs, and ICEs. There are also two constructed GI datasets based on whole-genome comparison [15], [27] (Table 1), which were used as training datasets for machine learning methods. But the scale of these datasets is still not large enough, and their reliability has not been verified by convincing biological evidence.

## 3 GI prediction methods

In spite of the above challenges, previous methods have made considerable progress in GI prediction. They usually use two most indicative features of the horizontal origin of GIs: biased sequence composition and sporadic phylogenetic distribution. Based on the two features, these methods roughly fall into two categories: *composition-based methods* and *comparative genomics-based methods*[17].

**Fig. 2:**
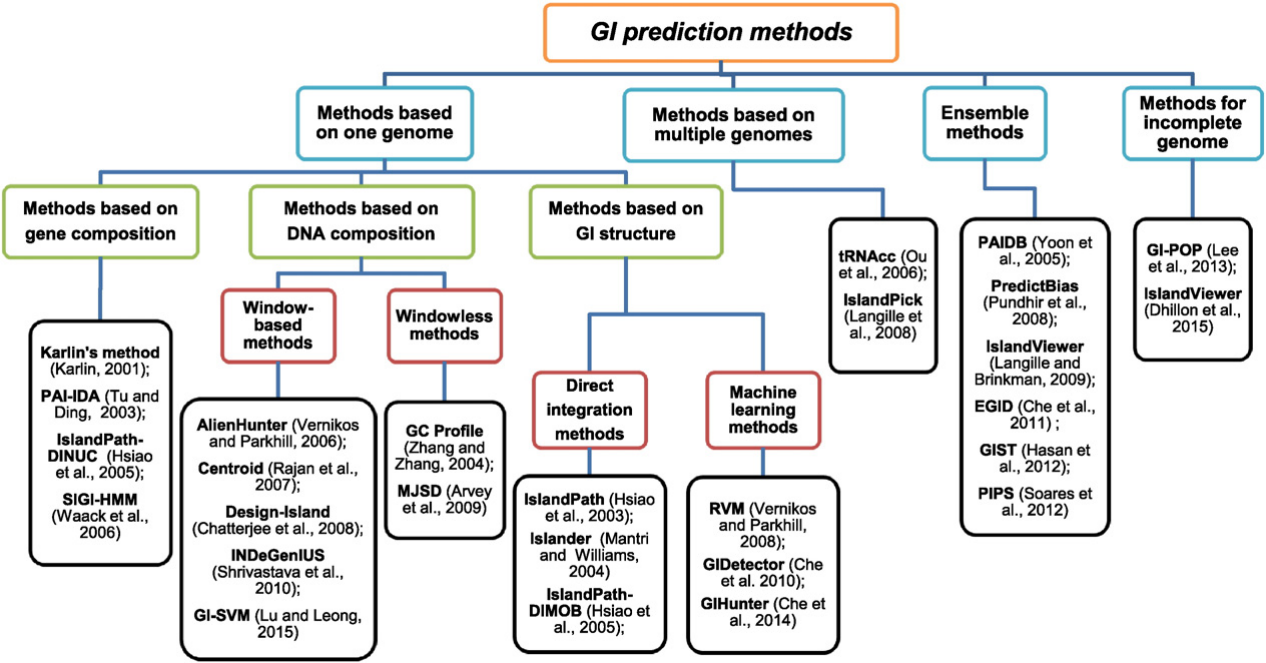
The hierarchical overview of computational methods for predicting genomic islands which are discussed in this paper.

For ease of discussion, we categorize GI prediction methods into two large groups based on the number of input genomes: *methods based on one genome* and *methods based on multiple genomes*. Methods in the former group are often composition based, while methods in the latter group are usually comparative genomics-based. We also include *ensemble methods* which combine different kinds of methods and *methods for incomplete genomes* which predict GIs in draft genomes (in the form of contigs or scaffolds rather than complete whole genome sequence). Fig. 2 shows an overview of the methods included in this paper. For reference, we list available programs which are discussed under each category in Table 2.

### 3.1 Methods based on one genome

Most methods based on one genome utilize sequence composition to identify GIs, but several methods based on GI structural characteristics have also been developed. According to the units for measuring genome composition, composition-based methods can be divided into *methods at the gene level* and *methods at the DNA level*. In the following sub-sections, we present the basic idea of composition-based methods before discussing methods at the gene and DNA level separately.

The major assumption of composition-based methods is that mutational pressures and selection forces acting on the microbial genomes may result in species-specific nucleotide composition [52]. Thus, a laterally transferred region may show *atypical composition* which is distinguishable from the average of the recipient genome. Under this assumption, most compositional methods try to choose certain sequence characteristics as discrimination criteria to measure the compositional differences. Several features have been shown to be good criteria, including *GC content*, *codon usage*, *amino acid usage*, and *oligonucleotide* (*k-mer*) *frequencies*[53]. Based on these criteria, single-threshold methods are often adopted for GI prediction. The atypicality of each gene or genomic region is measured by a score derived from the comparison with the average of the whole genome via similarity measures. The genes or genomic regions with scores below or above a certain threshold (either predefined or dynamically computed) are supposed to be atypical. The consecutive atypical genes or genomic regions are finally merged to get candidate GIs.

#### 3.1.1 Methods based on gene sequence composition

Methods based on gene sequence composition are often designed to detect LGT, or laterally transferred genes [54], and only a few methods are specifically developed to detect GIs. The methods for LGT detection can be utilized to identify GIs by combing clusters of laterally transferred genes, but they are supposed to be less sensitive, since some genes inside a GI may not show atypicality to allow the whole GI being captured. Here we mainly discuss specific methods for GI detection.

Some GI detection methods combine multiple discrimination criteria, such as Karlin's method [55] and PAI-IDA [30]. Karlin's method and PAI-IDA predict GIs and PAIs by evaluating multiple compositional features (*GC content*, *dinucleotide frequencies*, *codon usage*, and *amino acid usage*). Karlin's method is a single-threshold method, while PAI-IDA uses iterative discriminant analysis. Both methods use a sliding window to scan the genome, and sequences or genes inside each window are used for computation.

Other methods use only a single discrimination criterion, such as IslandPath-DINUC [40], [56] and SIGI-HMM [31]. IslandPath-DINUC uses a single-threshold method to predict GIs as multiple consecutive genes with only *dinucleotide bias*. SIGI-HMM predicts GIs and putative donor of laterally transferred genes based solely on the *codon usage bias* of individual gene. As an extension of SIGI [57], an earlier method based on scores derived from codon frequencies, SIGI-HMM substitutes the previous heuristic method with Hidden Markov Model (HMM) to model the laterally transferred genes and native genes as different states.

Methods based on gene sequence composition are generally easy to implement and apply. But what they indeed find are compositionally atypical genomic regions in terms of certain criteria. So there are many false positives and false negatives. Native regions may easily be detected as false positives owing to their atypical composition for reasons other than LGT, such as highly expressed genes [58]. At the same time, ameliorated GIs [52] or GIs originated from genomes with similar composition may not be detected. But the false positives can be reduced by eliminating well-known non-GIs. For example, by filtering out putative highly expressed genes based on codon usage, SIGI-HMM was reported to have the highest precision in a previous evaluation [27].

For methods performing comparisons with the genomic average, laterally transferred regions may contaminate the genome and reduce the accuracy of predictions [59]. Furthermore, the predicted boundaries of GIs are not precise, since the boundaries between laterally transferred genes and native genes can be compositionally ambiguous [54]. Additionally, these methods at the gene level require reliable gene annotations. Thus, they may not be applied to newly sequenced genomes, which have no or incomplete annotations.

#### 3.1.2 Methods based on DNA sequence composition

The increase of newly sequenced genomes without complete annotations necessitates GI prediction based on DNA sequences alone. Without the aid of gene boundaries, the large genome has to be segmented by other measures. According to genome segmentation approaches, methods based on DNA sequence composition can be classified into two major kinds: *window-based methods* and *windowless methods*.

Window-based single-threshold methods are commonly used for GI detection. These methods use a sliding window to segment the whole genome sequence into a set of smaller regions. There are several representative programs, including AlienHunter [32], Centroid [33], INDeGenIUS [35], Design-Island [34] and GI-SVM [36]. The major differences among them are in: the size of the sliding window, the choice of the discrimination criterion and similarity measure, and the determination of the threshold.

Both AlienHunter and GI-SVM use a fixed-size overlapping window of fixed step size. AlienHunter is the first program for GI detection on raw genomic sequences. It measures segment atypicality via relative entropy based on interpolated variable order motifs (IVOM). The threshold can be obtained by either k-means clustering or standard deviation (when there are fewer samples). GI-SVM is a recent method using either fixed or variable order k-mer frequencies. It detects atypical windows via one-class SVM with spectrum kernel. An automatic threshold can be obtained from one dimensional k-means clustering.

Centroid partitions the genome by a non-overlapping window of fixed size. The average of k-mer frequency vectors for all the windows is seen as the centroid. Based on the Manhattan distances from each frequency vector to the centroid, outlier windows are selected by a threshold derived from standard deviation. INDeGenIUS is a method similar to Centroid. But it uses overlapping windows of fixed size and computes the centroid via hierarchical clustering.

Design-Island is a two-phase method utilizing k-mer frequencies. It incorporates statistical tests based on different distance measures to determine the atypicality of a segment via pre-specified thresholds. In the first phase a variable-size window is used to obtain initial GIs, whereas in the refinement phase a smaller window of fixed size is used to scan over these putative GIs for getting final GI predictions.

Some of these methods are designed to alleviate the problem of genome contamination. Design-Island excludes the initially obtained putative GIs when computing parameters for the entire genome in the second phase. GI-SVM measures the atypicality of all the windows simultaneously via one-class SVM, and only some windows contribute to the genomic signature.

To deal with the imprecise GI boundaries that result from a large step size, AlienHunter uses HMM to further localize the boundaries between predicted GIs and non-GIs. But most other programs do not consider this issue.

The few windowless methods mainly include GC Profile [37], [60] and MJSD [39].

GC Profile is an intuitive method to calculate global GC content distribution of a genome with high resolution. The abrupt drop in the profile indicates the sharp decrease of GC content and thus the potential presence of a GI. This method was later developed into a web-based tool which is used for analyzing GC content in genome sequences [38]. However, other features have to be used together with GC Profile for GI prediction due to the poor discrimination power of GC content.

MJSD is a recursive segmentation method based on Markov Jensen-Shannon divergence (MJSD) measure. The genome is recursively cut into two segments by finding a position where the sequences to its left and to its right have statistically significant compositional differences. Subsequently, each segment is compared against the whole genome to check its atypicality via a predefined threshold.

Methods based on DNA sequence composition have the similar advantages and disadvantages as methods based on gene sequence composition.

Specifically, window-based methods can be highly sensitive with appropriate implementations. For example, AlienHunter was reported to have the highest recall in previous evaluation [27], and GI-SVM was recently shown to have even higher sensitivity than AlienHunter [36]. But their precisions are quite low due to the limited input information. They are also inherently incapable of identifying the precise boundaries between regions with compositional differences [39].

In contrast, windowless methods can delineate the boundaries between GIs and non-GIs more accurately [39]. GC Profile has successfully discovered a few reliable GIs in several genomes [60]. But it seems subjective to access the abruptness of jump in the GC profile, and only GIs with low GC content can be detected. MJSD is better at predicting GIs of size larger than 10 kb [39], but the procedure to determine segment atypicality still suffers from the contamination of the whole genome.

#### 3.1.3 Methods based on GI structure

The presence of compositional bias is usually not sufficient to assure the foreign origin of putative GIs. Thus, it is necessary to develop methods based on multiple GI-related structural features. According to the approaches of integrating different features, methods based on GI structure can be divided into *direct integration methods* and *machine learning methods*.

The direct integration methods adopt a series of filters to get more reliable GIs. But some integrated features are only used for validation, since it is difficult to systematically use them for prediction given the extreme GI structural variation. There are mainly two representative programs: IslandPath [40] and Islander [24].

IslandPath is the first program integrating multiple features (*GC bias*, *dinucleotide bias*, *the presence of tDNAs* and *mobility-related genes*) to aid GI detection. But IslandPath only annotates and displays these features in the whole genome, leaving it to the user to decide whether a region is a GI or not. Based on these computed features, a GI can be identified as multiple consecutive genes with both *dinucleotide bias* and *the presence of mobility-related genes* (IslandPath-DIMOB) [56].

Islander incorporates a method to accurately detect tDNA-borne GIs. Islander seeks specific tDNA signature to find candidate GIs. Several filters are used to exclude potential false positives, such as regions without integrase genes. Recently, the filtering algorithms are refined via incorporating more precise annotations available now [29].

Several machine learning approaches based on constructed GI datasets have been proposed, including Relevance Vector Machine (RVM) [15], GIDetector [41], and GIHunter [42]. The major differences among them are in the choices of training datasets, GI-related features, and learning algorithms.

RVM is the first machine learning method to study structural models of GIs. It is based on the datasets constructed from comparative genomics methods. Eight features of each genomic region are used to train GI models: *IVOM score*, *insertion point*, *GI size*, *gene density*, *repeats*, *phage-related protein domains*, *integrase protein domains* and *non-coding RNAs*.

GIDetector utilizes the same features and training datasets as RVM, but it implements decision tree based ensemble learning algorithm. GIHunter uses the similar algorithm as GIDetector, but adopts slightly different features and datasets. *GI size* and *repeats* are replaced by *highly expressed genes* and *average intergenic distance*. The training datasets are replaced by IslandPick datasets. The predictions of GIHunter for thousands of microbial genomes are available online at http://www5.esu.edu/cpsc/bioinfo/dgi/index.php.

Methods utilizing GI structure can generate more robust predictions. For example, the high reliability of GIs inserted at tDNA sites leads to very few false positives in the predictions from Islander [29]. But these methods depend on accurate identification of multiple related features, such as tRNA genes, mobility-related genes, and virulence factors.

Direct integration methods are straightforward, but many GIs may be filtered out due to the lack of certain features. For example, IslandPath-DIMOB was shown to have very low recall in spite of high accuracy and precision [27].

Conversely, machine learning approaches can systematically integrate multiple GI features to improve GI prediction. This can be partly reflected by the high recall and precision of GIHunter [42]. However, the performance of supervised methods is closely related to the quality of training datasets.

### 3.2 Methods based on several genomes

Methods based on several genomes detect GIs based on their sporadic phylogenetic distribution. They compare multiple related genomes to find regions present in a subset but not all the genomes. The comparison procedure often involves analyzing results from sequence alignment tools [17], such as local alignment tool BLAST [61], and whole-genome alignment tool MAUVE [62].

BLAST and MAUVE can be used to find unique strain-specific regions (GI candidates), whereas MAUVE can also be used to find conserved regions. For example, Vernikos and Parkhill performed genome-wide comparisons via all-against-all BLAST, and then applied manual inspection to find reliable GIs for training GI structural models [15]. They also differentiated gene gain from gene loss via a maximum parsimony model obtained from MAUVE alignments. Despite the tediousness of manual analysis, there are only two automatic methods based on several genomes: tRNAcc [43] and IslandPick [27].

The tRNAcc method utilizes alignments from MAUVE to find GIs between a conserved tRNA gene and a conserved downstream flanking region across the selected genomes. It was later integrated into MobilomeFINDER [63], an integrative web-based application to predict GIs with both computational and experimental methods. Complementary analysis is also incorporated in tRNAcc to provide additional support, including GC Profile, strain-specific coding sequences derived from BLAST analysis, and dinucleotide differences. But appropriate genomes to compare have to be selected manually.

To facilitate genome selection, IslandPick builds an all-against-all genome distance matrix and utilizes several cut-offs to select suitable genomes to compare with the query genome, making it the first completely automatic comparative genomics method. The pairwise whole-genome alignments are done by MAUVE to get large unique regions in the query genome. After being filtered by BLAST to eliminate genome duplications, these regions are considered as putative GIs.

Due to the inaccuracies of composition-based methods, methods based on several genomes are preferred if there are appropriate genomes for comparison [27]. But uncertainties still exist in their predictions. Firstly, the results are dependent on the genomes compared with the query genome [27]. Secondly, it is hard to distinguish between gene gain via LGT and gene loss [23]. Thirdly, genomic rearrangements can cause difficulties in accurate sequence alignments [62]. In addition, the applications of methods based on several genomes are limited, since the genome sequences of related organisms may not be available for some query genomes.

### 3.3 Ensemble methods

Different kinds of methods often predict non-overlapping GIs [17] and complement each other [39]. To make the best of available methods, ensemble methods have been proposed to combine different methods.

One way of combination is to merge the predictions from multiple programs. This approach is implemented in IslandViewer [44] and EGID [47]. IslandViewer is a web-based application combining three programs: SIGI-HMM, IslandPath-DIMOB, and IslandPick. It provides the first user-friendly integrated interface for visualizing and downloading predicted GIs. Newer versions of IslandViewer include further improvements [45], [46], such as improving efficiency and flexibility, incorporating additional gene annotations, and adding interactive visualizations. But the underlying integration method is mainly a union of predictions from individual programs. Unlike IslandViewer, EGID uses a voting approach to combine predictions from five programs: Alienhunter, IslandPath, SIGI-HMM, INDeGenIUS, and PAI-IDA. A user-friendly interface for EGID is provided in the program GIST [48].

Another way of combination is to filter the predictions from one method by other methods. This approach is common for PAI prediction, since it is critical to utilize multiple features to discern PAIs from other GIs. Several PAI detection programs adopt this approach, including PAIDB [64], PredictBias [49] and PIPS [50]. These programs often combine composition-based methods, comparative genomics methods, and homology-based methods.

Both PAIDB and PredictBias firstly identify *putative GIs* based on compositional bias. For PAIDB, the *putative GIs* homologous to published PAIs (overlapping with PAI-like regions obtained from homology searches) are seen as candidate PAIs. SIGI-HMM and IslandPath-DIMOB are later integrated into PAIDB for GI predictions [28]. To overcome the dependency on known PAIs, PredictBias constructs a profile database of virulence factors (VFPD). If the *putative GIs* (or eight contiguous genes) have a pre-specified number of significant hits to VFPD, they are seen as *potential PAIs*. PredictBias also integrates comparative analysis to validate the *potential PAIs*.

PIPS integrates multiple available tools for computing PAI-associated features. It filters out the initial predictions from comparative genomics analysis via empirical logic rules on selected features (*GC content*, *codon usage*, *virulence factors* and *hypothetical proteins*).

Combining the predictions of several programs is supposed to perform better than individual programs. Actually, IslandViewer was shown to increase the recall and accuracy without much sacrifice of precision [17], and EGID was reported to yield balanced recall and precision [47].

The available ensemble methods are mostly characterized by user-friendly interfaces, but the combination procedures do not seem to be sophisticated enough. Some valuable predictions made by one method may be discarded in the ensemble method. For example, PredictBias was shown to have lower sensitivity and accuracy than PIPS on two bacterial strains [50], which reflects the effects of different integration strategies on the performances to some extent.

### 3.4 Methods for incomplete genomes

Thanks to low-cost high-throughput sequencing, an increasing number of microbial genomes are being sequenced. However, many of these genomes are in draft status. So there is a need to predict GIs in incomplete genomes. Currently, there are only two programs for this purpose: GI-GPS [51] and IslandViewer 3 [46]. Both programs firstly assemble the sequence contigs into a draft genome, and then use methods similar to those for predicting GIs in complete genomes.

GI-GPS is a component of GI-POP, a web-based application integrating annotations and GI predictions for ongoing microbial genome projects. GI-GPS uses an assembler within GI-POP for genome assembly. Then an SVM classifier with radial basis function kernel is applied to segments obtained from a sliding window of fixed size along the genome. The classifier is trained on IslandPick datasets and selected GIs from PAIDB. GI-GPS utilizes compositional features in model training to tolerate potential errors in the assembled genome. The predictions from the classifier are filtered by homologous searches to keep only sequences with MGE evidence. Then the boundaries of filtered sequences are refined by repeats and tRNA genes.

IslandViewer 3 maps the annotated contigs to a completed reference genome to generate a concatenated genome. Then it uses this single genome as input to the normal IslandViewer pipeline.

GI-GPS and IslandViewer 3 make it feasible to predict GIs for draft genomes. But they are still simplistic and limited. For example, IslandViewer 3 is restricted to the genome which has very few contigs and reference genomes of closely related strains of the same species [46]. Furthermore, it seems inappropriate to apply methods similar to those developed for complete genomes, since draft genome sequences do not have as high quality as whole genome sequences.

## 4 Summary and outlook

Since the discovery in microbial genomes, the importance of GIs has been gradually appreciated. Extensive research has demonstrated multiple GI-associated signatures, but these features show great variation in different genomes. Nevertheless, several of these features have been revealed to be effective in GI detection and applied in many computational methods, including compositional bias, structural markers and phylogenetically restricted distribution. Based on the input data, we classify these methods into four large groups, which are further divided into subgroups based on the features utilized or the methodology adopted. It should be noted that some methods may belong to multiple categories. For example, tRNAcc and GI-GPS can also be classified as ensemble methods.

In short, distinct kinds of methods detect GIs based on diverse features and assumptions, and thus generate predictions of different reliabilities. Methods based on gene or DNA composition of a single genome provide only rough estimations, since they usually take advantage of very limited information. Methods based on GI structure utilize multiple lines of evidence, and are supposed to be more reliable. But compositional or structural features in a single genome can only provide static information for GI prediction. Instead, methods based on several genomes can reveal genetic flux among closely related genomes and provide dynamic information [3]. Therefore, they can be more accurate. To get more comprehensive and reliable results, it seems desirable to use methods based on more evidence, such as ensemble methods and methods based on GI structure. This can be illustrated by the evaluations of some methods on the well-studied S. typhi CT18 genome (Table 3).

19 reference GIs were obtained from [39], excluding two GIs of size smaller than 5 kb. The predictions of each program were either downloaded from the corresponding website (IslandViwer (including the predictions from SIGI-HMM, IslandPath-DIMOB, and IslandPick), tRNAcc, GIHunter) or from running the program on local machine with optimal parameters (GI-SVM, EGID). The evaluation metrics (Recall, Precision, F1) were measured as those in [36]. All the relevant data and scripts can be found at https://github.com/icelu/GI_Prediction.

Although the sophistication and performance of GI prediction methods have been steadily improved, there is still room for further improvement. For instance, the precision and recall of current methods are still not high enough [43], suggesting the presence of many false negatives and false positives. This can be improved either by more advanced integration of multiple kinds of methods or refinement on a single kind of methods.

For GI prediction based on a single genome, machine learning methods may help. On one hand, DNA composition-based prediction can be seen as contiguous subsequence based anomaly detection [65], whose goal is to find anomalous contiguous subsequences significantly different from other subsequences in a long sequence. From this perspective, many computational approaches for outlier detection may be adapted for GI prediction. On the other hand, it seems feasible to apply more sophisticated supervised learning algorithms for structure-based GI prediction, since the accumulation of reliable GIs can provide a more solid basis for model training.

For GI prediction based on incomplete genomes, methods directly applied to sequence contigs without initial genome assembly may be developed. Despite the challenges in analyzing short sequences, there has been a method proposed to detect LGT in metagenomic sequences which consist of contigs from different species in an environment [66]. This approach may be inspiring for predicting GIs from the contigs directly.

## Figures and Tables

**Table 1:** The available datasets related to genomic islands.

Name	Feature	Availability
*Database*		
PAIDB [25], [28]	The only database including most reported PAIs and REIs	http://www.paidb.re.kr/about_paidb.php
Islander [24], [29]	Intended to be gold standard dataset for accurately mapped GIs	http://bioinformatics.sandia.gov/islander
ICEberg [26]	Providing comprehensive information about ICEs	http://db-mml.sjtu.edu.cn/ICEberg/

*Constructed dataset*		
RVM datasets [15]	331 GIs and 337 non-GIs from 37 bacteria of 3 genera	Not available
IslandPick datasets [27]	771 GIs and 3770 non-GIs from 118 bacteria of 12 orders	http://www.pathogenomics.sfu.ca/islandpick_GI_datasets/

**Table 2:** The summary of selected programs for predicting genomic islands.

Program	Form	Availability
*Methods based on gene composition of one genome*		
PAI-IDA [30]	Command line	Upon request
SIGI-HMM [31]	Graphical interface	https://www.uni-goettingen.de/en/research/185810.html

*Methods based on DNA composition of one genome*		
Window-based methods		
AlienHunter [32]	Command line	http://www.sanger.ac.uk/resources/software/alien_hunter
Centroid [33]	Command line	Upon request
Design-Island [34]	Command line	http://www.isical.ac.in/~rchatterjee/Design-Island.html
INDeGenIUS [35]	Command line	Upon request
GI-SVM [36]	Command line	https://github.com/icelu/GI_Prediction
Windowless methods		
GC Profile [37], [38], [60]	Web-based	http://tubic.tju.edu.cn/GC-Profile
MJSD [39]	Command line	http://cbio.mskcc.org/~aarvey/mjsd/

*Methods based on GI structure of one genome*		
Direct integration methods		
IslandPath [40]	Web-based	http://www.pathogenomics.sfu.ca/islandpath/
Machine learning methods		
GIDetector [41]	Command line	http://www5.esu.edu/cpsc/bioinfo/software/GIDetector
GIHunter [42]	Command line	http://www5.esu.edu/cpsc/bioinfo/software/GIHunter

*Methods base on multiple genomes*		
tRNAcc [43]	Web-based	http://db-mml.sjtu.edu.cn/MobilomeFINDER/
IslandPick [27]	Command line	http://www.pathogenomics.sfu.ca/islandviewer/download/

*Ensemble methods*		
IslandViewer [44], [45], [46]	Web-based	http://www.pathogenomics.sfu.ca/islandviewer
EGID [47]	Command line	http://www5.esu.edu/cpsc/bioinfo/software/EGID
GIST [48]	Graphical interface	http://www5.esu.edu/cpsc/bioinfo/software/GIST
PredictBias [49]	Web-based	http://www.bioinformatics.org/sachbinfo/predictbias.html
PIPS [50]	Command line	http://www.genoma.ufpa.br/lgcm/pips

*Methods for incomplete genome*		
GI-POP [51]	Web-based	http://gipop.life.nthu.edu.tw

**Table 3:** The comparisons of selected programs for predicting genomic islands on S. typhi CT18 genome.

Program	Category	Recall	Precision	F1
GI-SVM	Methods based on DNA composition	0.895	0.446	0.596
EGID	Ensemble methods	0.779	0.535	0.634
SIGI-HMM	Methods based on gene composition	0.241	0.556	0.337
IslandViewer	Ensemble methods	0.654	0.670	0.662
GIHunter	Methods based on GI structure	0.827	0.676	0.744
IslandPath-DIMOB	Methods based on GI structure	0.553	0.788	0.650
tRNAcc	Methods based on several genomes	0.286	0.993	0.444
IslandPick	Methods based on several genomes	0.060	1.000	0.114

The evaluations were based on 19 reference GIs obtained from [39], excluding two GIs of size smaller than 5 kb. The predictions of each program were either downloaded from the corresponding website (IslandViwer (including the predictions from SIGI-HMM, IslandPath-DIMOB, and IslandPick), tRNAcc, GIHunter) or from running the program on local machine with optimal parameters (GI-SVM, EGID). The evaluation metrics (Recall, Precision, F1) were measured as those in [36]. All the relevant data and scripts can be found at https://github.com/icelu/GI_Prediction.
